# Splenic Artery Embolization and Splenectomy for Spontaneous Rupture of Splenic Hemangioma and Its Imaging Features

**DOI:** 10.3389/fcvm.2022.925711

**Published:** 2022-06-03

**Authors:** Jia-Li Lin, Can Lin, Han-Lu Wang, Shao-Jie Wu, Yi Tang, Chang Shun Yang, Jie-Wei Luo, Wu Chi, Zhu-Ting Fang

**Affiliations:** ^1^Fujian Provincial Hospital, Shengli Clinical Medical College of Fujian Medical University, Fuzhou, China; ^2^Department of Radiology, Fuzhou Second Hospital, Fuzhou, China; ^3^Department of Interventional Radiology, Fujian Provincial Hospital, Fuzhou, China; ^4^Emergency Department, Fujian Provincial Hospital, Fuzhou, China

**Keywords:** splenic disease, splenic rupture, splenic artery, artery embolization, splenectomy

## Abstract

**Background:**

Spontaneous splenic rupture (SSR) is a rare, often life-threatening, acute abdominal injury that requires immediate diagnosis and early treatment. SSR is mainly treated surgically or conservatively. A few cases of interventional embolization for SSRs have been reported.

**Case Presentation:**

A 30-year-old male patient complaining mainly of left upper abdominal pain underwent emergency abdominal computed tomography (CT) and showed enlargement of the spleen with a massive mixed-density shadow approximately 10.0 × 8.0 × 12.5 cm in size. The boundary was unclear and showed obvious progressive enhancement. Considering the intrasplenic tumor lesions with rupture and hemorrhage, the possibility of vascular tumors was high, with intraperitoneal blood and fluid accumulation. Digital subtraction angiography of the splenic arteriography and embolization of the ruptured splenic artery branches were performed. Postoperative hemoglobin progressively decreased, inflammatory indicators, such as white blood cell counts, procalcitonin (PCT), and C-reactive protein (CRP) were significantly increased, and 2 days after embolization, the patient developed severe hypoxemia, shock, pulmonary edema, and acute respiratory distress syndrome. CT re-examination 9 days after embolization showed reduced lesion absorption. After stabilization of the condition, splenectomy was performed, and postoperative platelet count increase, anticoagulant improvement, and discharge were observed. Postoperative pathological examination revealed extensive hemorrhage and necrosis, vascular tissue with abnormal hyperplasia in the surrounding area, vascular tissue in the bleeding area and outer wall (elastic fiber staining +), and local myofibroblast hyperplasia. Immunohistochemistry showed actin (SM +) and Ki67 (10% +).

**Conclusion:**

SSR caused by splenic hemangioma is rare, and the choice between surgical treatment or splenic artery embolization remains dependent on the patient's hemodynamic stability and imaging findings.

## Introduction

Spontaneous splenic rupture (SSR) is a rare acute condition that is often life-threatening, with an associated mortality rate of 12.2%, SSR due to SH is even rarer, accounting for only 0.7% of the etiology of SSR (1). Nontraumatic splenic rupture was first reported by Rokitansky in a leukemia patient in 1891, and apparently normal spleen rupture was reported by Atkinson in 1874. Rupture of the spleen in a normal spleen was defined as spontaneous type, and rupture of the spleen in patients with underlying disease was defined as pathological rupture (2). SSR requires immediate diagnosis and urgent early treatment to ensure patient survival. The treatment of SSR is mainly surgical or conservative. Few cases of interventional embolization for spontaneous splenic ruptures have been reported. In this report, we describe a rare case of spontaneous splenic hemangioma (SH) rupture treated with emergency embolization followed by splenectomy and review of the imaging features of different periods.

## Case Presentation

**Figure 1:**
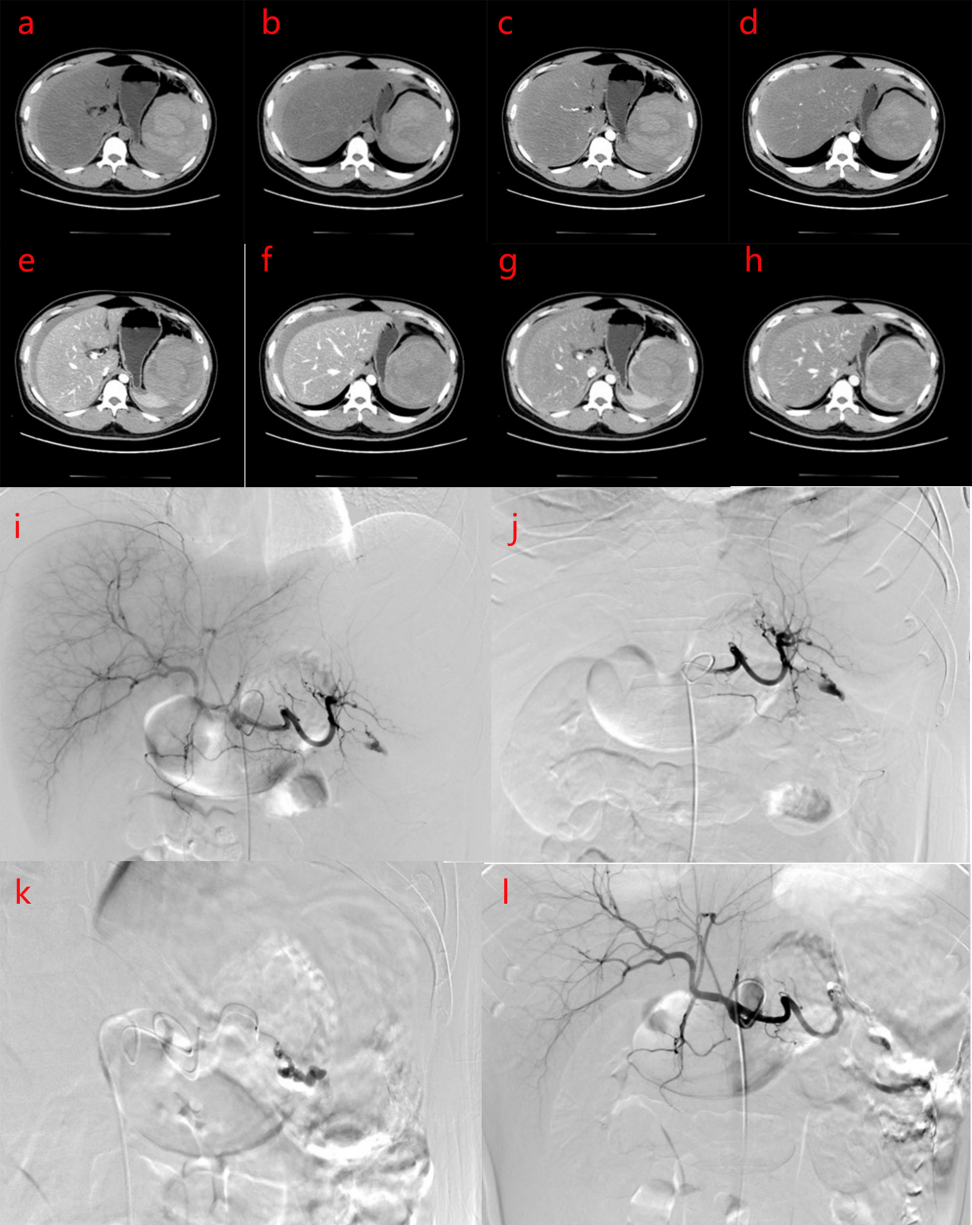
**(a–h)** Plain computed tomography (CT) scan and enhancement of the upper abdomen CT before intervention shows that the density of liver parenchyma is significantly reduced, and the shadow of intrahepatic vessels shows relative density on plain CT scan. The spleen is enlarged, and there is a mixed density shadow in the spleen with a size of about 10.0 × 8.8 × 12.5 cm. The plain CT value is about 11–67 HU, the boundary is unclear, and the enhanced CT shows partly obvious progressive lesion enhancement. Patchy and slightly high-density shadows were observed around the spleen, liver, and abdominal space, and the CT value is about 44–71 HU. The clinical impression is neoplastic lesions with rupture and hemorrhage, which may be vascular tumors. Intraperitoneal accumulation of blood and fluid. Severe fatty liver. **(a,b)** PLAIN CT scans; **(c,d)** Arterial phase of CT; **(e,f)** Portal veinphase of CT; **(g,h)** Delayphase of CT. **(i–l)** Digital subtraction angiography (DSA) and interventional embolization of the spleen. DSA showed splenic artery rupture and bleeding, abdominal fluid accumulation.

A 30-year-old male patient presented with sudden persistent left upper abdominal pain without obvious cause 11 h before admission, with progressive aggravation accompanied by nausea and vomiting of stomach contents, fatigue, and sweating. Emergency abdominal computed tomography (CT) showed that the spleen was enlarged, with a massive mixed density shadow approximately 10.0 × 8.0 × 12.5 cm in size. The boundary was unclear, and showed obvious progressive enhancement. Considering the intrasplenic tumor lesions with rupture and hemorrhage, the possibility of vascular tumors was high, with intraperitoneal blood and fluid accumulation. Severe fatty liver is shown in Figures 1a–h. Laboratory tests showed a white blood cell count of 24.4 × 10^9^/L (normal 3.5–9.5 × 10^9^/L), red blood cell count of 3.81 × 10^12^/L (normal 4.3–5.8 × 10^12^/L), and hemoglobin of 118 g/L(normal 130–175 g/L). Blood gas analysis showed a pH of 7.246, pCO_2_ of 21.2, residual base of 16.3, standard bicarbonate of 12.2, and actual bicarbonate of 9.7. Tests showed an activated partial thromboplastin time of 26.9 s (normal 23.3–32.5 s), fibrinogen of 0.93 g/L (normal 1.8–3.5 g/L), and d-dimer of 0.85 mg/L FEU (normal 0–0.55 mg/L FEU), while other coagulation indexes were generally normal. Biochemical indicators showed albumin levels of 38 g/L (normal 40–55 g/L), total bilirubin of 10.27 μmol/L (normal value ≤ 23.0 μmol/L), aspartate aminotransferase of 41 U/L (normal value 15–40 U/L), creatinine of 167 μmol/L (normal 40–135 μmol/L), potassium of 5.2 mmol/L (normal 3.5–5.3 mmol/L), and sodium of 130 mmol/L (normal 137–147 mmol/L), while other routine biochemical indicators were generally normal. The patient's blood pressure decreased to 85/54 mmHg, accompanied by oliguria. The patient's clinical manifestations and laboratory indicators suggested hypovolemic shock, indicating active anti-shock rehydration and emergency interventional surgery.

Under local anesthesia, the right femoral artery was punctured and intubated, and a 5FRH catheter was inserted. The tip was placed in the celiac trunk for selective splenic artery digital subtraction angiography (DSA). Transcatheter embolization of microspheres (300–500 and 500–700 μm), medical glue, ultra-liquid iodized oil (Guerbet, Paris, France), and α-*n*-butyl cyanoacrylate (Compont, Beijing, China) were mixed in a 3:1 ratio, and the ruptured splenic artery branch was embolized. Angiography revealed that the main splenic artery was clearly displayed, the branches in the spleen were not clearly displayed, the branches of the splenic artery in the lower part of the spleen were ruptured, the contrast medium was exuded, and the ruptured splenic artery branches were blocked after embolization with microspheres and medical glue.The peripheral defect of the spleen was approximately 40%, and the overall operation was smooth (Figures 1i–l).

After embolization, the patient's heart rate was still fast, fluctuating at around 120 beats/min, and the hemoglobin level gradually decreased to 63 g/L. Considering the possibility of bleeding, we decided to perform laparoscopic splenectomy 1 day after embolization. On the way to the operating room, owing to the decrease in peripheral oxygen saturation, the patient was transferred to the intensive care unit. Emergency bronchoscopy showed a large amount of thin sputum, blood gas analysis showed respiratory failure, and bedside ultrasound showed a large number of B lines in both lungs. Pulmonary edema or acute respiratory distress syndrome (ARDS) were considered, ventilator-assisted respiratory was performed, with high positive end-expiratory pressure (PEEP) to prevent alveolar collapse and improve oxygenation. Infusion of 300 mL fresh frozen plasma was performed twice, and O rh-positive white suspended erythrocytes were transfused 2 u, 1.5 u, and 1 u in stages, and the condition improved after treatment of acid production, fluid replacement, and anti-infection.

**Figure 2:**
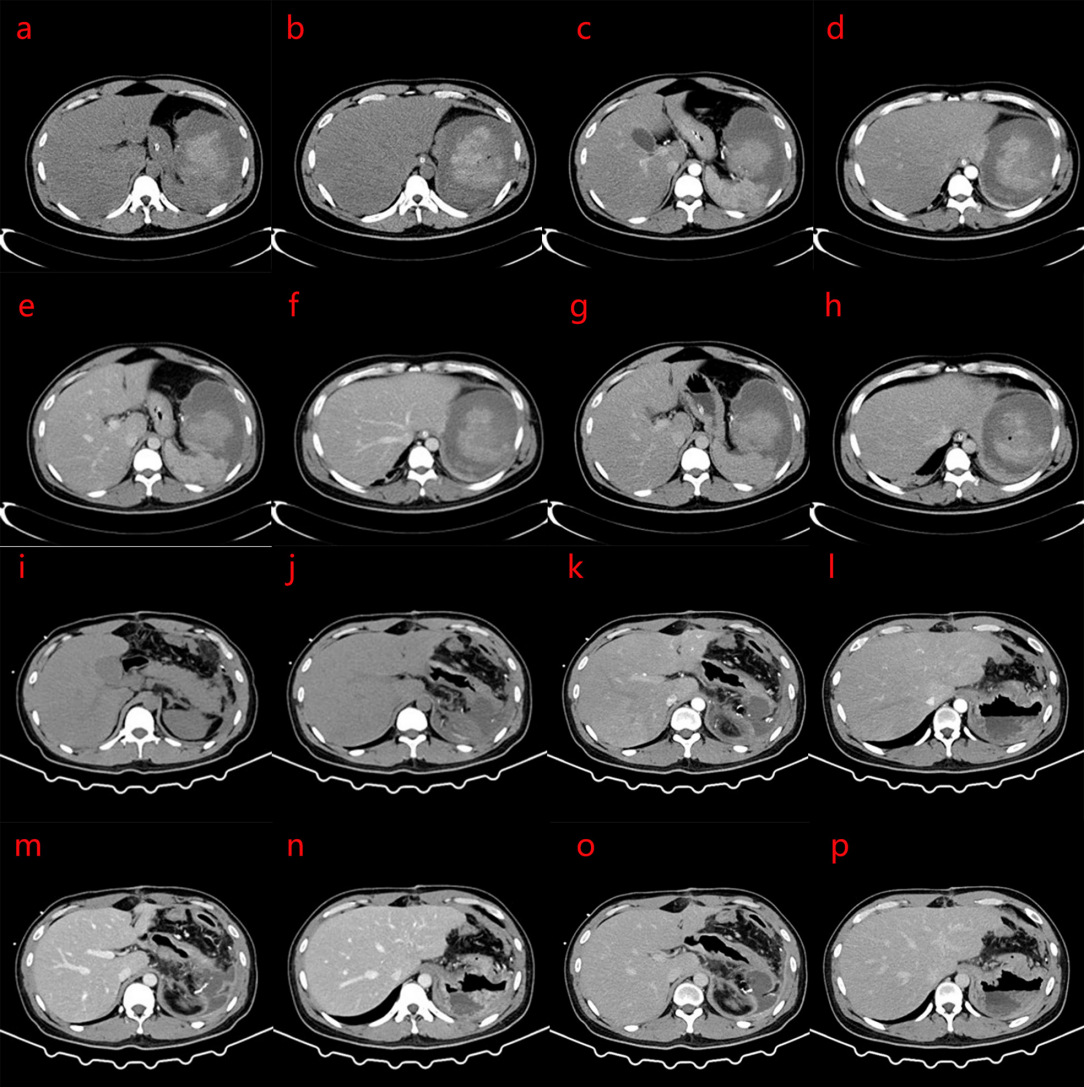
**(a–p)** Computed tomography (CT) scan 9 days after splenic artery embolization: the spleen is enlarged, with strip-like and spot-like dense shadows and gas accumulation in the operation area; a mass mixed density shadow is seen, and the internal high density mass was about 8.0 × 7.3 × 7.8 cm. The CT value of the plain scan is about 11–67 HU. The enhanced scan part shows progressive enhancement. The edge is surrounded by low-density shadows, the wall is slightly enhanced, the boundary with the adjacent spleen is unclear, and this extends to the paracolic space. The rest of the spleen parenchymal part of the enhancement is reduced, showing irregular flakes or wedges. Patchy and cord-like high-density shadows are seen in the remaining adjacent abdominal fat spaces, with poorly defined boundaries, and the left renal anterior fascia is thickened. No obvious space-occupying lesions are found in the pancreas. The density of liver parenchyma is significantly reduced. The clinical impression is a change after splenic artery embolization, which is better than before, and severe fatty liver. **(i–p)** Changes after splenectomy, patchy and cord-like dense shadows are seen in the operation area, and the adjacent abdominal fat space, with poorly defined borders, and scattered patchy low-density shadow. The larger size mass was approximately 5.3 × 5.6 cm, with part of it encapsulated. Part of the edge of the enhanced scan shows mild enhancement, and the anterior fascia of the left kidney is thickened. **(a,b,i,j)** CT plain scans; **(c,d,k,l)** Arterial phase of CT; **(e,f,m,n)** Portal vein phase of CT; **(g,h,o,p)** Delay phase of CT.

The activated partial thromboplastin time was 11.6 s, fibrinogen was 3.36 g/L, and D-dimer was 9.92 mg/L FEU. Albumin was 36 g/L, potassium was 4.5 mmol/L, sodium was 135 mmol/L, white blood cell count was 13.1 × 10^9^/L (normal 3.5–9.5 × 10^9^/L), hemoglobin levels were 74 g/L, and the platelet count was 716 × 10^9^/L (normal, 125–350 × 10^9^/L). Nine days after embolization, the CT scan showed that the spleen was enlarged, and a small amount of gas was observed in the operation area. A massive mixed density shadow was visible, with a high density shadow of about 8.0 × 7.3 × 7.8 cm. The plain CT value was about 11–67 HU, and the enhanced scan part appeared to have progressive enhancement, with the edge surrounded by low-density shadow, the wall was slightly enhanced, and the boundary with the adjacent spleen was unclear and extended to the paracolon space, considering for tumor lesions with rupture, hemorrhage, and a slightly higher-density shadow than before. The enhancement of the remaining splenic parenchyma was reduced, and showed irregular flakes or wedges, which were infarcts. Patchy and cord-like high-density shadows were observed in the remaining adjacent abdominal fat spaces, with poorly defined boundaries, and the left renal anterior fascia was thickened (Figures 2a–p). Severe fatty liver is shown in Figures 2a–g.

**Figure 3:**
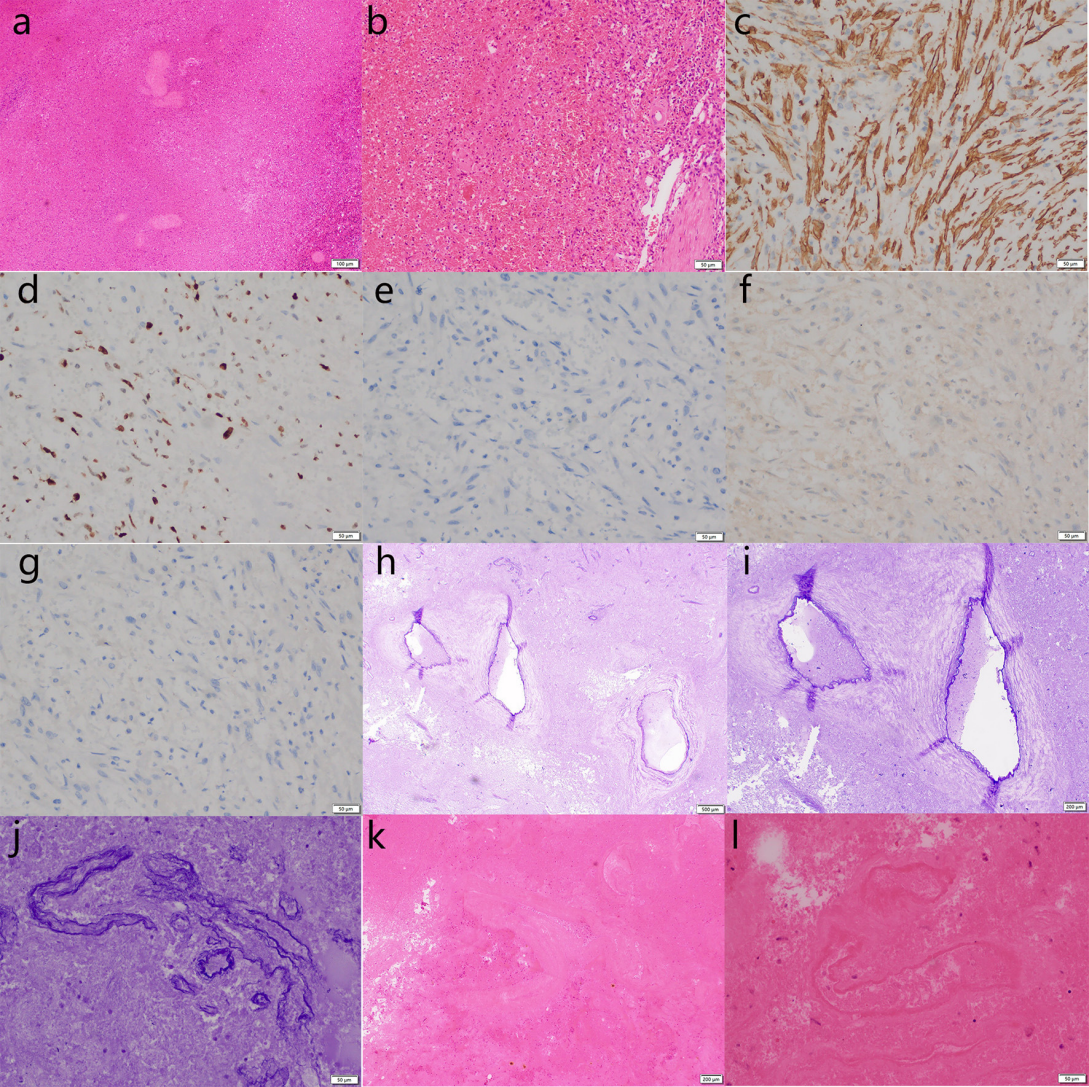
Pathological biopsy of the splenectomy specimen shows large hemorrhage and necrosis under microscope, and abnormally proliferated vascular tissue around the hemorrhage area [hematoxylin & eosin staining; **(a)** ×100; **(b)**, ×200; **(k)**, ×40; **(l)**, ×200]. Vascular tissue (elastic fiber staining +), blood vessel wall tissue (elastic fiber staining +), and local hyperplasia of myofibroblastic cells are shown [**(h)**, ×20; **(i)**, ×40; **(j)** ×200]. Immunohistochemistry showed actin [SM +, **(c)** ×200], Ki67 [10% +, **(d)** ×200), ALKp80 [–, **(e)** ×200], melanA [–, **(f)** ×200], HMB45 [–, **(g)** ×200]. The above findings are consistent with a splenic hemangioma with ruptured hemorrhage and necrosis.

Laparoscopic splenectomy was performed 12 days after the embolization. A splenectomy specimen was obtained that was cut into the upper pole of the spleen and close to the capsule. A cystic solid mass measuring 9 × 8 × 6 cm was found, containing blood clots. The cut surface was gray-red and spongy, and bleeding was observed after the extrusion. Postoperative pathological diagnosis (splenic tumor): a splenectomy specimen was examined under a microscope, with large hemorrhage and necrosis, surrounding vascular tissue with abnormal hyperplasia of the structure, vascular tissue in the bleeding area (elastic fiber staining +), vascular wall tissue in the bleeding outer wall (elastic fiber staining +), and local myofibroblast proliferation. Immunohistochemistry showed actin (SM) (+), HMB45 (–), melanA (–), ALKp80 (–), and Ki67 (10% +). Special dyeing: elastic fiber dyeing (+). Combined with imaging, immunohistochemistry, and special staining, the diagnosis of SH with ruptured hemorrhage and necrosis was confirmed (Figure 3).

Starting 5 days after embolization, the platelet count increased progressively, reaching a maximum of 716 × 10^9^/L. After splenectomy, the platelet count continued to increase, reaching a maximum of 1,165 × 10^9^/L. Antiplatelet therapy with aspirin was administered. A CT scan performed 9 days after splenectomy showed post-splenectomy changes, and inflammatory exudation in the surgical area and adjacent abdominal fat space, combined with encapsulated effusion, and thickening of the anterior fascia of the left kidney.

The patient was discharged 10 days after splenectomy. At the 3 month follow-up visit, the patient was in good clinical condition and had no other complications.

The case study was approved by the Ethics Committee of Fujian Provincial Hospital, China (K2021-01-033).With the informed consent of patients and their families.

## Discussion

### SSR Due to SH

Splenic hemangioma, though rare, is the most common tumor in the spleen, and the second most common focal splenic lesion after simple splenic cysts, with an incidence of 0.02%−0.16% reported in a large number of autopsies. SH is characterized by excessive vascular proliferation. A total of 80% of SHs occur in asymptomatic individuals. Only a minority of patients have symptoms and signs directly related to SHs. Most SHs are discovered incidentally, or during the evaluation of other diseases, such as in surgery or imaging examinations (3, 4).

Splenic hemangioma appears as single or multiple lesions on CT scans, and is usually homogeneous, hypodense, or polycystic (5). It may contain calcifications and usually exhibits peripheral enhancement after intravenous contrast. Ultrasonography usually shows a round echogenic mass with or without cystic areas (6). Microscopically, most hemangiomas are spongy in nature, and consist of large interconnected, dilated, blood-filled spaces composed of a monolayer of cytologically bland endothelial cells interspersed with thin fibrous septa or splenic marrow tissue. Pure capillary structures are rare; however, many lesions contain varying proportions of cavernous and capillary components (7). Although SHs are benign lesions, they can rupture and cause intra-abdominal hemorrhage, resulting in a range of severe clinical symptoms (7).

Spontaneous splenic rupture is rare, with a mortality rate of 12.2%. The causes of SSR are roughly divided into six groups: neoplastic lesions (30.3%), infectious lesions (27.3%), immune, non-infectious lesions (20.0%), drug or treatment-related lesions (9.2%), mechanical lesions (6.8%), and SSR (6.4%) occurred in normal spleen. The most common causes are hematological malignancies (e.g., acute leukemia) and viral infections (e.g., cytomegalovirus infection) (1). Bellingham et al. (8) reported a case of sudden splenic rupture in an 8-year-old boy with acute lymphoblastic leukemia 9 days after chemotherapy. Spontaneous rupture of the spleen due to SH is even rarer, accounting for only 0.7% of the etiology of SSR (1). Rao et al. (9) reported the case of a 35-year-old man with recurrent epigastric and left flank pain and severe anemia due to a ruptured cavernous SH. Pathological findings also revealed granulomatous lesions consistent with tuberculosis in the rest of the spleen parenchyma. Norris et al. (10) reported the case of a 76-year-old man with a benign cavernous SH that spontaneously ruptured after thrombolysis due to myocardial infarction. Both of these patients underwent splenectomy.

### Treatment of SSR

Spontaneous splenic rupture treatment includes total splenectomy (84.1%), organ-sparing surgery (including splenoplasty and partial splenectomy) (1.2%), or conservative treatment (14.7%) (1). A third treatment option, splenic artery embolization (SAE), has only recently been reported (11–13).

The choice of treatment requires consideration of the following factors: etiology of the SSR, hemodynamic stability, volume of blood transfusion, patient status, degree of splenic injury, and volume of intraperitoneal bleeding (1). When SSR has a non-malignant etiology and can be properly monitored, conservative approaches should be attempted. Compared to splenectomy, interventional therapy has the advantage of preserving functional spleen tissue and enabling its immune function to play a role (8, 13), especially in younger patients and in those with infectious diseases. In addition, this avoids the risk of overwhelming postsplenectomy infection (OPSI) after splenectomy (14). Splenectomy improves patient survival and avoids new bleeding; in cases where the etiology is unknown, histological examination of the spleen can provide additional etiological information (1, 8, 15). However, SAE is an option before surgery. Preoperative SAE can provide patients with the dual benefits of hemostasis and spleen preservation, which can not only reduce blood flow and achieve rapid hemostasis, but also reduce the risk of major bleeding during surgery.

### Reconsider of the Case

We reconsidered the diagnosis and treatment of this case, and found that patients with stable or unstable hemodynamics often need to choose different treatment options. It is generally considered that patients with hemodynamic instability should be prioritized for emergency splenectomy. However, successful SAEs have also been reported in patients with hemodynamic instability. Kim et al. (13) reported the case of a Korean malaria patient with splenic rupture, unstable hemoglobin levels, and blood pressure. Splenic angiography revealed an irregular contour at the lower margin of the lateral margin. Embolization was performed at the distal level of the splenic artery by using seven coil springs. After embolization, the subcapsular hematoma of the spleen was reduced, and the hemoglobin level remained normal. SAE is recommended in patients with hemodynamic stability in an angiographically positive setting. SAE can also be used to reduce the likelihood of delayed splenic rupture (12).

There are reports of successful treatment of unruptured hemangioma by SAE (16), but patients with spontaneous rupture of splenic hemangioma and hemodynamic instability has rarely been reported. The patient had severe abdominal pain, hemodynamic instability and hypovolemic shock on admission. In order to stop bleeding and stabilize the patient's condition as soon as possible, embolic microspheres and medical glue were used for emergency embolization, so as to try spleen-preserving therapy. After embolization, the heart rate and blood pressure of the patients were improved, but the hemoglobin decreased gradually, indicating that SAE did not completely stop bleeding. Persistent bleeding may still occur after SAE treatment (11%), and some patients can be re-treated with SAE, and half of them need splenectomy (17). The efficiency of hemostasis may be related to the location of embolism. For the rupture of spleen caused by tumor, only embolizing the ruptured blood vessels of splenic artery branches may still lead to wound bleeding and continuous decrease of hemoglobin. There are also reports suggesting the use of splenic artery main embolization combined with splenic branch embolization to enhance hemostatic efficiency. SAE is a conservative spleen-preserving technique, which selectively embolizes part of the splenic circulation, causing local ischemia and infarction, weakening the phagocytic function of the spleen, and achieving the surgical effect equivalent to partial splenectomy (18). In view of the important role of spleen in immunity, splenectomy should be used cautiously. However, compared with splenectomy, SAE has the risk of rebleeding, and splenectomy is still needed in the end. Swaid (11) and Spittle (19) have similar reports of splenectomy after SAE failure.

In addition, the patient in this report had recurrent fever after SAE, and inflammatory indicators, such as white blood cell count, procalcitonin (PCT), and C-reactive protein (CRP), indicated splenic embolism syndrome, and 2 days after embolization, the patient developed restlessness, chest tightness, and shortness of breath. Peripheral oxygen saturation continued to decrease to a minimum of 34%, respiration was 40 beats/min, heart rate was 170 beats/min, and systolic blood pressure dropped to 70 mmHg. Therefore, severe hypoxemia and shock should be considered. Ultrasonography showed a large number of B-lines in both lungs, and it was highly possible to consider pulmonary edema or ARDS. The patient was given ventilator-assisted respiration, high PEEP to prevent alveolar collapse, and with improved oxygenation, blood transfusion, anti-infection, and other treatments, the patient's condition was stabilized. SAE may lead to a variety of postoperative complications, such as splenic abscess, atraumatic rupture, and post-embolic syndrome. These complications not only affect the clinical efficacy of SAE, but may also lead to death (18). Major complications occurred in 19% of patients, while minor complications occurred in 23%. Another study reported severe complications in 15 patients (3.7%) with SAE, including abscess, dyspnea caused by massive pleural effusion, ascites, pneumonia, pulmonary embolism, portal vein thrombosis and liver failure, and four cases (1%) died (20). It is generally recommended that broad-spectrum antibiotics be given before and after SAE to be safe (21).

### Limitations and Strengths

In conclusion, SSR caused by SH is rare, and the indications for surgery and SAE depend on the patient's hemodynamic stability and imaging findings (22). Because of the important role of the spleen in immunity, spleen-sparing surgery is preferable, where possible. Although rebleeding and reoperation may occur after embolization, vascular embolization can quickly stop bleeding and reduce the risk of major bleeding.

This case is a new exploration for the treatment of spontaneous splenic rupture with hemodynamic instability. However, the follow-up time of this case was not long enough, and the long-term effect of this treatment on the patient remains to be further observed.

### Recommendations for Future Research

Studies involving more patients could provide more evidence of the benefits and safety of the therapy.

### Clinical Implications for Health Managers and Policymakers

Reducing the cost of interventional surgery and including it in relevant medical policies can reduce the cost of medical care for patients and bring benefits to them.

## Data Availability Statement

The raw data supporting the conclusions of this article will be made available by the authors, without undue reservation.

## Ethics Statement

The studies involving human participants were reviewed and approved by the Ethics Committee of Fujian Provincial Hospital. The patients/participants provided their written informed consent to participate in this study. Written informed consent was obtained from the individual(s) for the publication of any potentially identifiable images or data included in this article.

## Author Contributions

J-LL, YT, H-LW, and CL performed the acquisition, analysis, and interpretation of the clinical data. J-LL and CL drafted the manuscript. S-JW and CY provided critical revision of the manuscript. J-WL, WC, and Z-TF designed and supervised the study. All authors read and approved the final manuscript.

## Funding

This work was supported by Fujian Province Natural Science Fund Project (2020J011096 and 2020J011064), the Special Research Foundation of Fujian Provincial Department of Finance (No. 2020-822 and 2021-917), and Outstanding Youth Project of Fujian Provincial Hospital (2014YNQN31).

## Conflict of Interest

The authors declare that the research was conducted in the absence of any commercial or financial relationships that could be construed as a potential conflict of interest.

## Publisher's Note

All claims expressed in this article are solely those of the authors and do not necessarily represent those of their affiliated organizations, or those of the publisher, the editors and the reviewers. Any product that may be evaluated in this article, or claim that may be made by its manufacturer, is not guaranteed or endorsed by the publisher.

## Abbreviations

SSR: spontaneous splenic rupture
SH: splenic hemangioma
SAE: splenic artery embolization
CT: computed tomography
PCT: procalcitonin
CRP: C-reactive protein
CTA: computed tomography angiography
DSA: digital subtraction angiography
OPSI: overwhelming postsplenectomy infection
ARDS: acute respiratory distress syndrome.
